# Direct Synthesis and Characterization of Hydrophilic Cu-Deficient Copper Indium Sulfide Quantum Dots

**DOI:** 10.1021/acsomega.3c09531

**Published:** 2024-04-04

**Authors:** Amanda Richardson, Jan Alster, Petro Khoroshyy, Jakub Psencik, Jan Valenta, Roman Tuma, Kevin Critchley

**Affiliations:** † Astbury Centre for Structural Molecular Biology, University of Leeds, Leeds LS2 9JT, U.K.; ‡ School of Molecular and Cellular Biology, Faculty of Biological Sciences, University of Leeds, Leeds LS2 9JT, U.K.; § Department of Chemical Physics, Faculty of Mathematics and Physics, Charles University, Prague 121 16, Czech Republic; ∥ School of Physics and Astronomy, Faculty of Engineering and Physical Sciences, University of Leeds, Leeds LS2 9JT, U.K.; ⊥ Faculty of Science, University of South Bohemia, Ceske Budejovice 370 05, Czech Republic

## Abstract

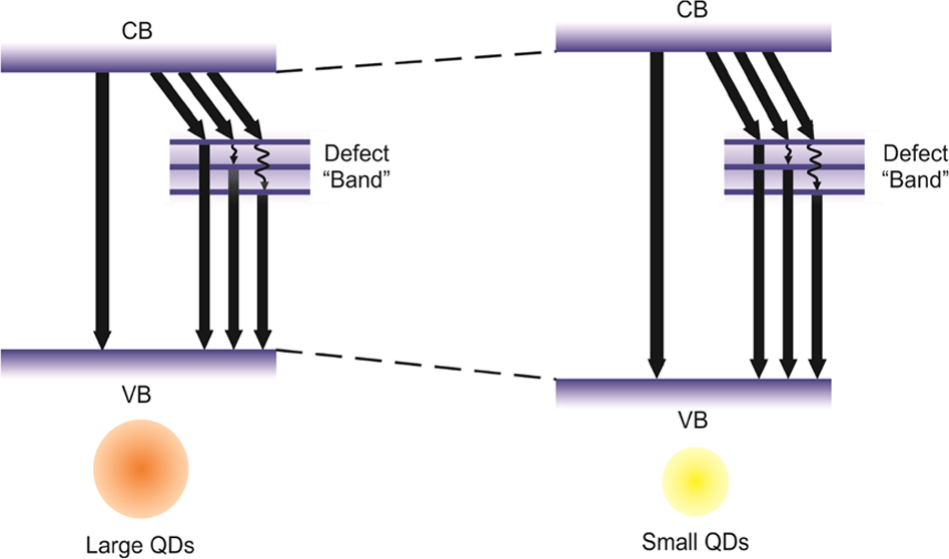

Copper indium sulfide (CIS) nanocrystals constitute a promising alternative to cadmium- and lead-containing nanoparticles. We report a synthetic method that yields hydrophilic, core-only CIS quantum dots, exhibiting size-dependent, copper-deficient composition and optical properties that are suitable for direct coupling to biomolecules and nonradiative energy transfer applications. To assist such applications, we complemented previous studies covering the femtosecond–picosecond time scale with the investigation of slower radiative and nonradiative processes on the nanosecond time scale, using both time-resolved emission and transient absorption. As expected for core particles, relaxation occurs mainly nonradiatively, resulting in low, size-dependent photoluminescence quantum yield. The nonradiative relaxation from the first excited band is wavelength-dependent with lifetimes between 25 and 150 ns, reflecting the size distribution of the particles. Approximately constant lifetimes of around 65 ns were observed for nonradiative relaxation from the defect states at lower energies. The photoluminescence exhibited a large Stokes shift. The band gap emission decays on the order of 10 ns, while the defect emission is further red-shifted, and the lifetimes are on the order of 100 ns. Both sets of radiative lifetimes are wavelength-dependent, increasing toward longer wavelengths. Despite the low radiative quantum yield, the aqueous solubility and long lifetimes of the defect states are compatible with the proposed role of CIS quantum dots as excitation energy donors to biological molecules.

## Introduction

Ternary I–III–VI element group quantum dots (QDs) have attracted increasing attention.^1^ In particular, copper indium sulfide (CIS) nanoparticles are emerging as a promising alternative to cadmium- and lead-based counterparts. In addition to lower toxicity,^2,3^ CIS nanoparticles exhibit quantum confinement, broad excitation bands, and good chemical and optical stability that are necessary for biological and biomedical applications.^4−10^ The high extinction coefficient of CIS,^11^ combined with a bulk direct band gap of ∼1.5 eV^12^ and tunable emission from visible to near-infrared wavelengths,^2,12,13^ makes CIS QDs ideally suited to lighting, solar energy harvesting, and bioimaging applications. The large Stokes shift exhibited by CIS nanoparticles leads to lower self-reabsorption and consequently less loss, further enhancing their suitability for energy applications.^14^ Recent improvements in synthesis have demonstrated a variety of techniques suitable for CIS QD synthesis, including solvothermal methods,^15−22^ thermolysis,^17,23−29^ photochemical decomposition,^30^ and hot injection.^28,31−34^ In addition, methods have been developed for controlling the nanoparticle crystal phase.^15,16,35^ However, most of these methods yield hydrophobic nanoparticles that are not suitable for bioimaging or coupling to biomolecules without further modification.

For example, the hydrophobic CIS QDs need to be modified by ligand exchange, organic polymeric coating, lipid coating, or inorganic silica coatings.^36−38^ Silica coatings are generally too thick for efficient energy transfer (e.g., electron or Forster-type). Similarly, polymer coatings are effective for phase transfer, but the effective hydrodynamic diameter increases substantially, and the control over the surface chemistry is decreased. Lipid encapsulation causes a degree of clustering of CIS QDs and the properties of the individual CIS QD are compromised.^39^ Ligand exchanges of alkanethiol to an ω-alkanethiol are not efficient, in comparison to oleic acid to thiol headgroup exchanges,^40^ due to the former having the same headgroup affinity.^41^ Furthermore, QD ligand exchanges usually result in a photoluminescence quantum yield (PLQY) decrease. Therefore, to enable the CIS synthesis to be performed with precise control over the organic surface chemistry and the thickness of the organic layer and enable the possibility of conjugation without surface modification, our approach was to synthesize the CIS/ZnS QDs in the presence of two ω-alkanethiols with hydrophilic tail groups (–PEG–Biotin and –OH (1:50)) so that the QDs would be dispersed in polar solvents without further modification.

PLQY of core–shell CIS QDs can be as high as 80–96%.^42,43^ However, in contrast to the narrow emission profiles of band edge emitting materials such as CdSe,^44^ CIS QDs exhibit a large Stokes shift and a relatively broad emission band, suggesting a significant contribution from intraband defect recombination pathways.^24,42,45,46^ In addition, for larger CIS nanoparticles approaching the Bohr exciton radius, the long wavelength side of the photoluminescence (PL) spectral peak extends further than that allowed by a band edge transition in the bulk material. Furthermore, the PLQY of Cu-deficient CIS QDs, with their associated high density of copper vacancy defects, is greater than that of an equivalent stoichiometric sample,^47,48^ indicating that defects play a significant role in CIS QD emission. Simulations of thin films suggest that ternary I–III–VI materials are highly defect tolerant with a band gap stable even against high degrees of off-stoichiometry,^49−51^ though the size dependence of this has not been investigated for materials specially confined in three dimensions.

Several studies using the photoluminescence (PL) lifetime have provided further evidence for this defect-driven emission and sought to identify a possible mechanism. Average PL lifetimes for CIS nanoparticles (100–300 ns), and I–III–VI nanoparticles in general, are longer than their II–VI counterparts (10–30 ns).^51^ PL decays for CIS nanoparticles cannot be described by a single exponential,^28^ and triple exponential decays with lifetimes of 4–12, 28–60, and 140–300 ns were used to describe the kinetics. These components were attributed to the existence of three types of recombination: intrinsic band exciton recombination, surface-related recombination, and defect-related recombination, with emission from the longest lifetime defect states accounting for 40–80% of the PL emission. Recent results favor an exciton self-trapping (or free-to-bound) mechanism in which a delocalized electron recombines with a hole localized at a copper ion.^42,48^ This model also explains the observed large Stokes shift.

Donor–acceptor pair (DAP) recombination has been suggested as the origin of the long-lived defect-related emission. For DAP recombination to take place, coupling of the conduction band to an emitting donor state with either radiative or nonradiative energy transfer is required. While previous studies have shown that emission on excitation is instantaneous (at least on the nanosecond time scale), an ultrafast population transfer between the conduction band and “electron-deficient” donor states remains possible.^52^ Studies of CIS, and similar CuInSe, materials indicate that due to their low energy of formation, defects consisting of antisites and copper vacancies (in the form of In_Cu_ + 2 V_Cu_, in Kröger–Vink notation) likely constitute the electron-deficient domain.^53−57^ Such defects likely exist as either isolated point defects, associated pairs, or clusters, depending on defect density.

In addition to this highly emissive long-lived channel, Li and co-workers^42^ observed a faster decaying component, which could be suppressed via surface coating with ZnS or CdS shells. Combined with the concomitant increase in PLQY, this suggests that the primary path of nonradiative decay is recombination through surface defect states. The higher PLQY makes the core–shell nanoparticles suitable for imaging and lighting applications. However, core nanoparticles are better suited for charge and energy transfer applications via direct coupling to other nanoparticles and photoactive molecules. One such application is light harvesting for photovoltaic and photochemical applications in which nanoparticles with tunable properties would augment and interface with biological light harvesting and photosynthetic systems. For such applications, a thorough understanding of the energetics and radiative as well as nonradiative decay pathways is critical. Likewise, these nanoparticles need to be soluble in aqueous buffers commonly used to dissolve biological molecules.

We report a novel method for the direct synthesis of high-quality hydrophilic nanoparticles with PLQY comparable to the best-reported values for core CIS nanoparticles^21,24,28,32,42,45,47,48,58,59^ and tunable size-dependent emission. This method yields nanoparticles with variable degrees of copper deficiency and allows us to systematically explore the role of defects in radiative and nonradiative recombination processes using time-resolved photoluminescence and transient absorption on a nanosecond time scale. In accord with previous studies,^28^ we identified two key recombination pathways that contribute to PL emission decay: band gap and defect-mediated recombination, respectively. We observed wavelength-dependent lifetimes for the nonradiative relaxation within the first excited band, while the defect states decayed with an approximately constant lifetime. Larger nanoparticle size led to a decrease in copper deficiency and a concomitant increase in PLQY. This is likely due to the reduced proportion of surface defects, which were suggested to mediate nonradiative recombination in comparison to volume defects associated with radiative recombination.^1,60^

## Results and Discussion

### Structure and Composition

**Figure 1:**
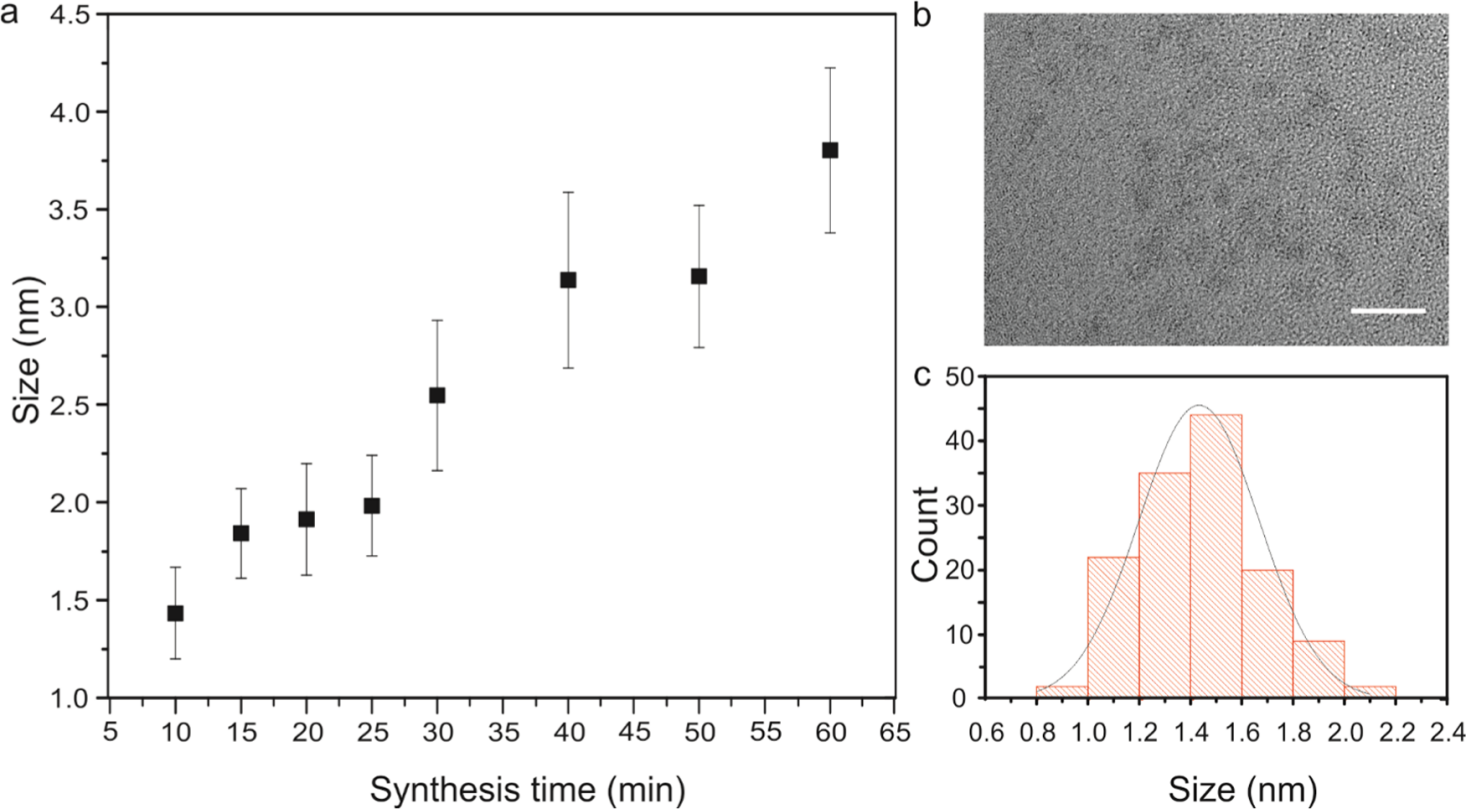
Nanoparticle size: (a) Nanoparticle diameter as a function of synthesis time determined by transmission electron microscopy (TEM), (b) sample TEM image used for sizing for a synthesis time of 10 min, bar: 5 nm, and (c) nanoparticle size distribution for 10 min synthesis time.

The aim of the synthesis was to produce hydrophilic copper-deficient CIS nanoparticles (see the Experimental section). During refluxing, a gradual color change from yellow through red to brown-black was observed, indicating nucleation and subsequent growth of the nanoparticle sizes. Cleaned nanoparticle samples were prepared for analysis. Transmission electron microscopy (TEM) was used to determine the size distribution (diameter) and morphology of the nanoparticles. Well-dispersed nanoparticles were observed in the TEM images (Figure 1b). The size distributions for each sample were fitted to normal distributions (Figure 1c). The full width at half-maximum (fwhm) of the distributions ranged from 0.2 to 0.5 nm. The mean size of the nanoparticles increased with synthesis time from 1.4 ± 0.2 nm (10 min) to 3.8 ± 0.4 nm (60 min, Figure 1a). Methods of optical spectroscopy were used to further characterize the nanoparticles and showed that the optimal synthesis time was between 15 and 30 min (see below).

**Figure 2:**
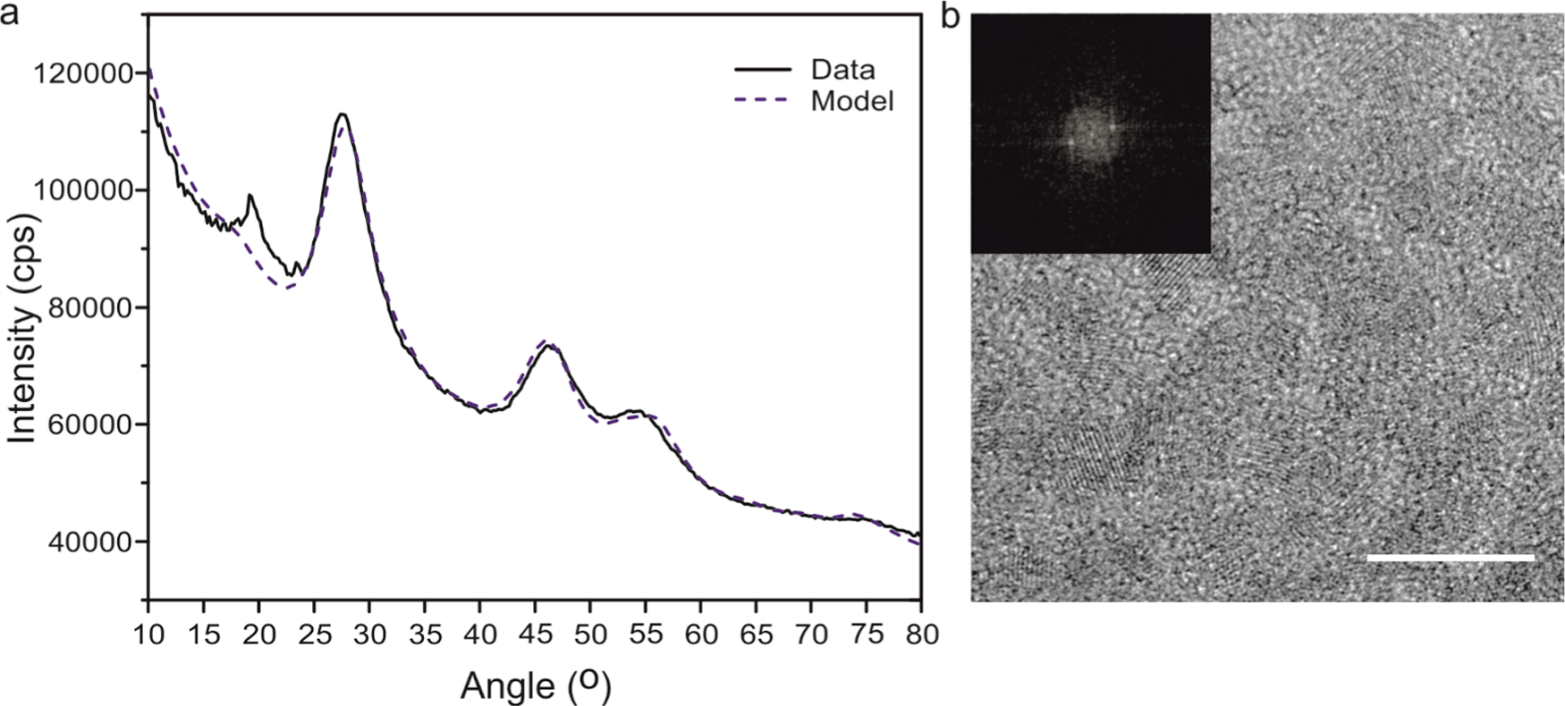
Structure: (a) X-ray diffraction (XRD) pattern for CIS nanoparticles (black line) in good agreement with reference data for CIS chalcopyrite (blue dashed line). (b) High-resolution transmission electron microscopy (HRTEM) image with clearly visible lattice spacings, bar: 5 nm. Inset: Fourier transform modulus of the image of lattice spacing, d = 0.31 nm, corresponding to the (112) crystal plane of chalcopyrite CIS.

Powder X-ray diffraction (XRD) patterns for the nanoparticles were consistent with reference data for the tetragonal chalcopyrite CIS phase (Figure 2a). High-resolution TEM images show 0.31 nm lattice fringes, which is consistent with the (112) planes of chalcopyrite CIS^61^ (Figure 2b). The combination of TEM and XRD suggests that the resultant nanoparticles are of the same structure as those prepared using dodecanethiol as a solvent and sulfur source.^11^

**Figure 3:**
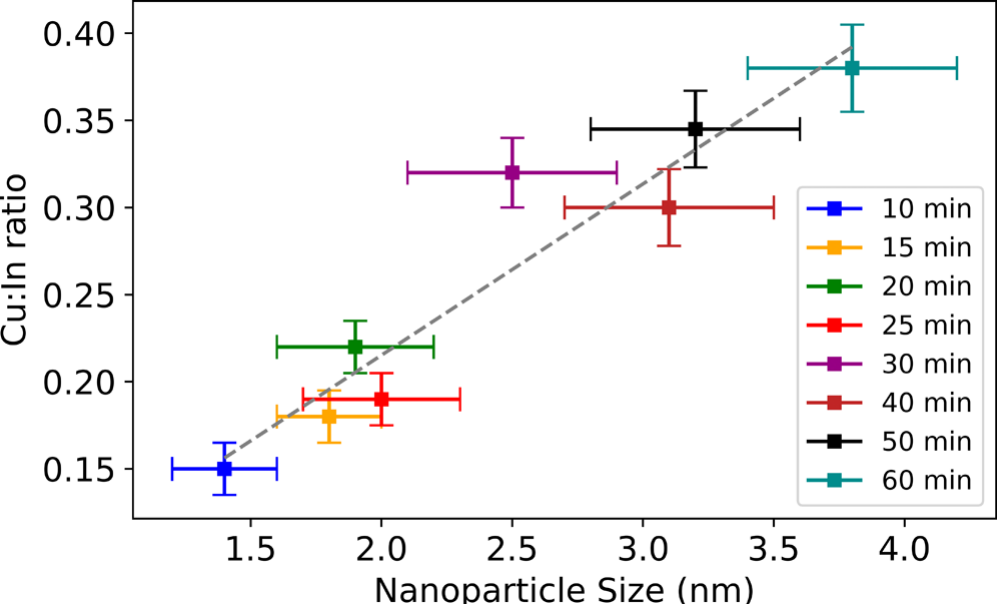
Composition: the dependence of the copper-to-indium ratio on nanoparticle size was determined by XPS. The dashed grey line is the line of best fit.

X-ray photoelectron spectroscopy (XPS) analysis was performed to determine the elemental composition of the nanoparticles. By measuring the relative area of the major XPS peaks for each element and correcting for sensitivity, the mean atomic composition was determined for an ensemble of particles drop-cast onto the surface. The analysis revealed the formation of highly copper-deficient nanoparticles with an increase in the Cu/In ratio observed with increasing size (Figure 3). While copper-deficient nanoparticles were expected as a result of the molar ratios used in the synthesis, a trend of increasing Cu/In with size was unexpected. This may be explained either by the annealing of Cu vacancies with increasing synthesis time or by an increase in available copper or moderation of relative reactivities with higher temperatures. Given that Cu deficiency decreases with decreasing surface-to-volume ratio for larger particles, it is likely that the vacancies are preferably localized within or close to the surface.

### Optical Properties

**Figure 4:**
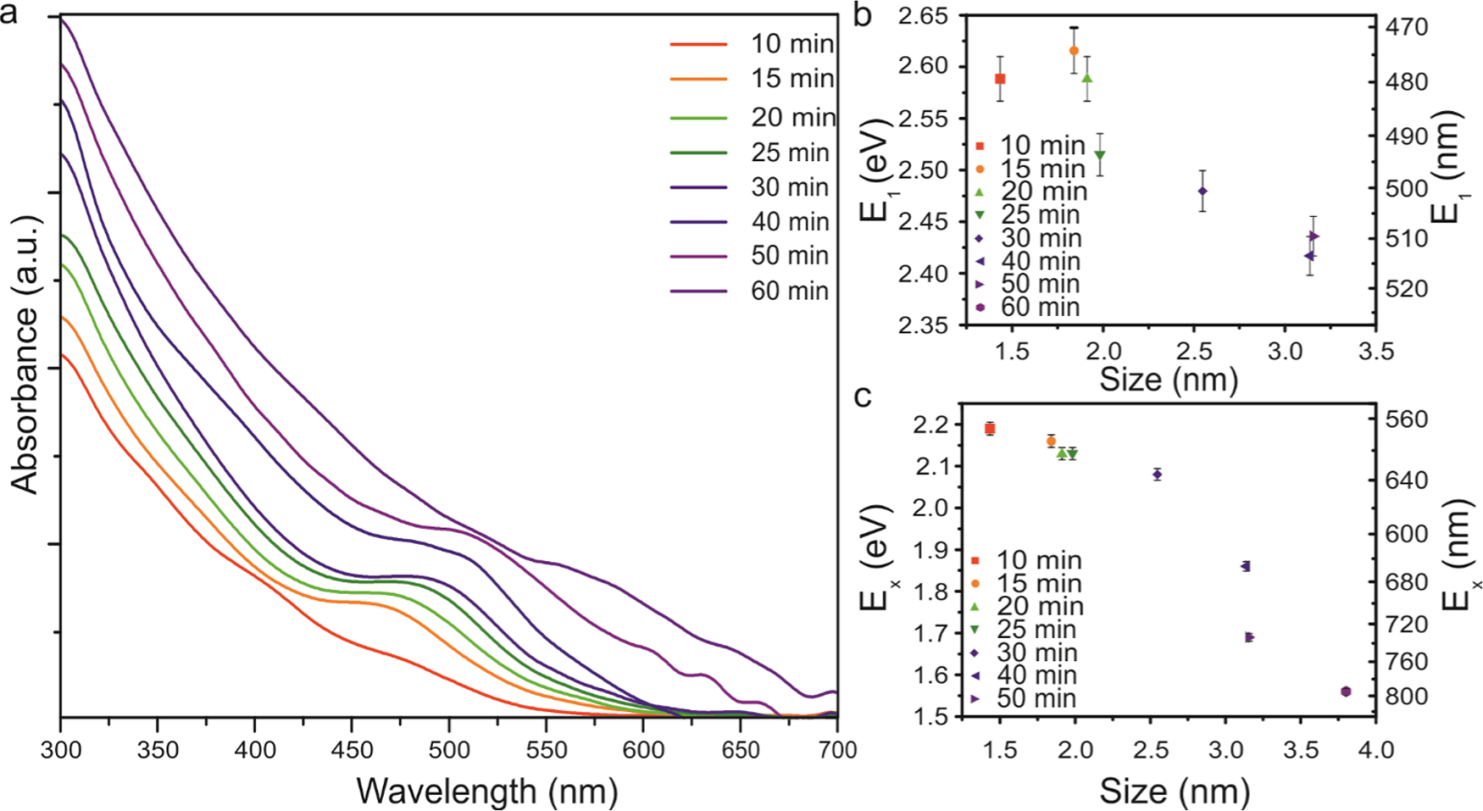
Optical properties: (a) Absorption spectra of CIS nanoparticles of different sizes. In contrast to previous studies, the absorption spectra of these particles show a distinct absorption feature. The position of this feature is significantly red-shifted with increasing synthesis time. (b) Size dependence of the energy of the first transition, E_1_, as determined from the second derivative of the absorption spectra. (c) Optical band gap energy E_*x*_ dependence on NP size.

The optical absorption spectra for the synthesized nanoparticles exhibit a broad band (or shoulder) and an absorption edge (Figure 4a). The absorption peak at around 450–500 nm is well discernible and of clear shape for the nanoparticles with synthesis times between 15 and 30 min, confirming that these are well formed. A shorter growing time is probably too short for the particles to form well. Aggregation (and subsequent phase separation) probably occurs for times longer than 30 min, since absorption bands are very broad, with features masked out by scattering. There is a marked red shift in the absorbance spectra with increasing synthesis time (Figure 4a) and particle size (Figure 4b,c). The shift reflects size-dependent quantum confinement of states within the nanocrystal and is consistent with previous observations.^28,62^ In contrast with previously observed spectra, the nanoparticles synthesized here exhibit a more pronounced absorbance band (shoulder) corresponding to the first excited band (E_1_). The energy difference of the optical band gap (E_*x*_) decreases with increasing particle size (Figure 4c), which is consistent with observations for other CIS synthesis methods.^62^ Additionally, the energy of the first transition also decreased with increasing particle size^11^ (Figure 4b).

**Figure 5:**
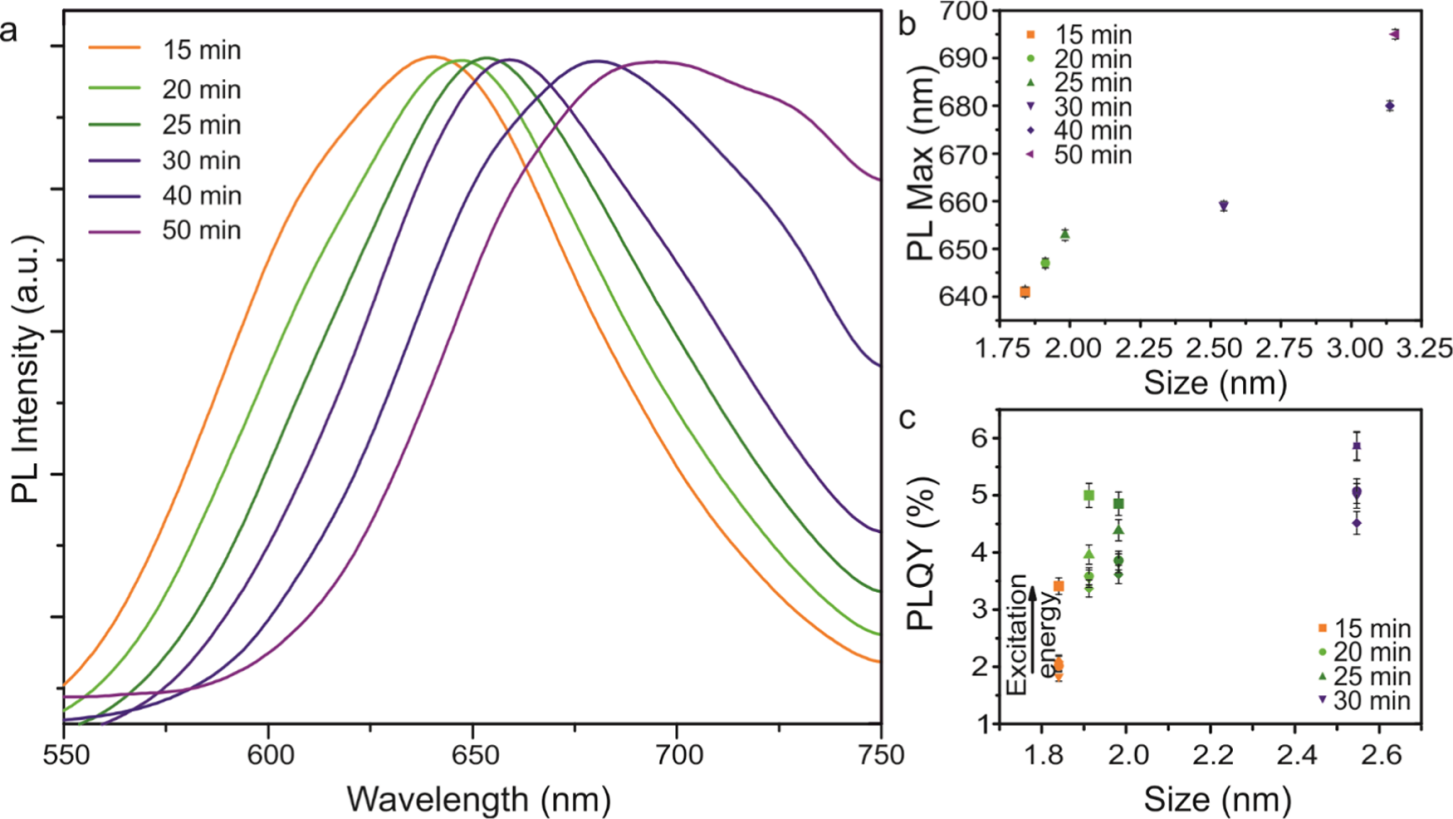
Photoluminescence: (a) Photoluminescence (PL) spectra for various sizes of quantum dots. A significant red shift in the position of the PL peak is observed with increasing particle size. (b) Dependence of PL peak position on nanoparticle size. (c) Photoluminescence quantum yield (PLQY) for nanoparticles between 1.84 and 2.55 nm at different excitation energy densities (intensities).

Figure 5 shows the PL spectra for copper-deficient CIS nanoparticles. A broad PL emission peak with a large Stokes shift was observed for all particles except those with the longest synthesis time (60 min), which exhibited no detectable PL. As concluded, based on visible absorption spectra, this is most likely a consequence of the phase separation of the larger particles. A red shift in the position of the PL maximum with increasing nanoparticle size was observed between ∼640 and 695 nm (Figure 5a,b).^1^ Again, the data indicate that the optimal synthesis time is between 15 and 30 min. These conditions lead to PL peaks of almost the same fwhm and maxima that shift to red regularly with synthesis time. PLQY measurements ranged from ∼2 to 6%, which is comparable with previously published values^21,24,28,32,42,45,47,48,58,59^ of core-only CIS QDs. Figure 5c shows that the PLQY increases with increasing particle size, indicating that the dominant nonradiative competing process is most likely mediated by surface Cu vacancies. Likewise, the PLQY increases with the deposited excitation energy density. The strong dependence of the absorbance and emission on the nanoparticle size demonstrates that the optical properties can be tuned by varying the synthesis time.

### Kinetics of Photoluminescence Decay

**Figure 6:**
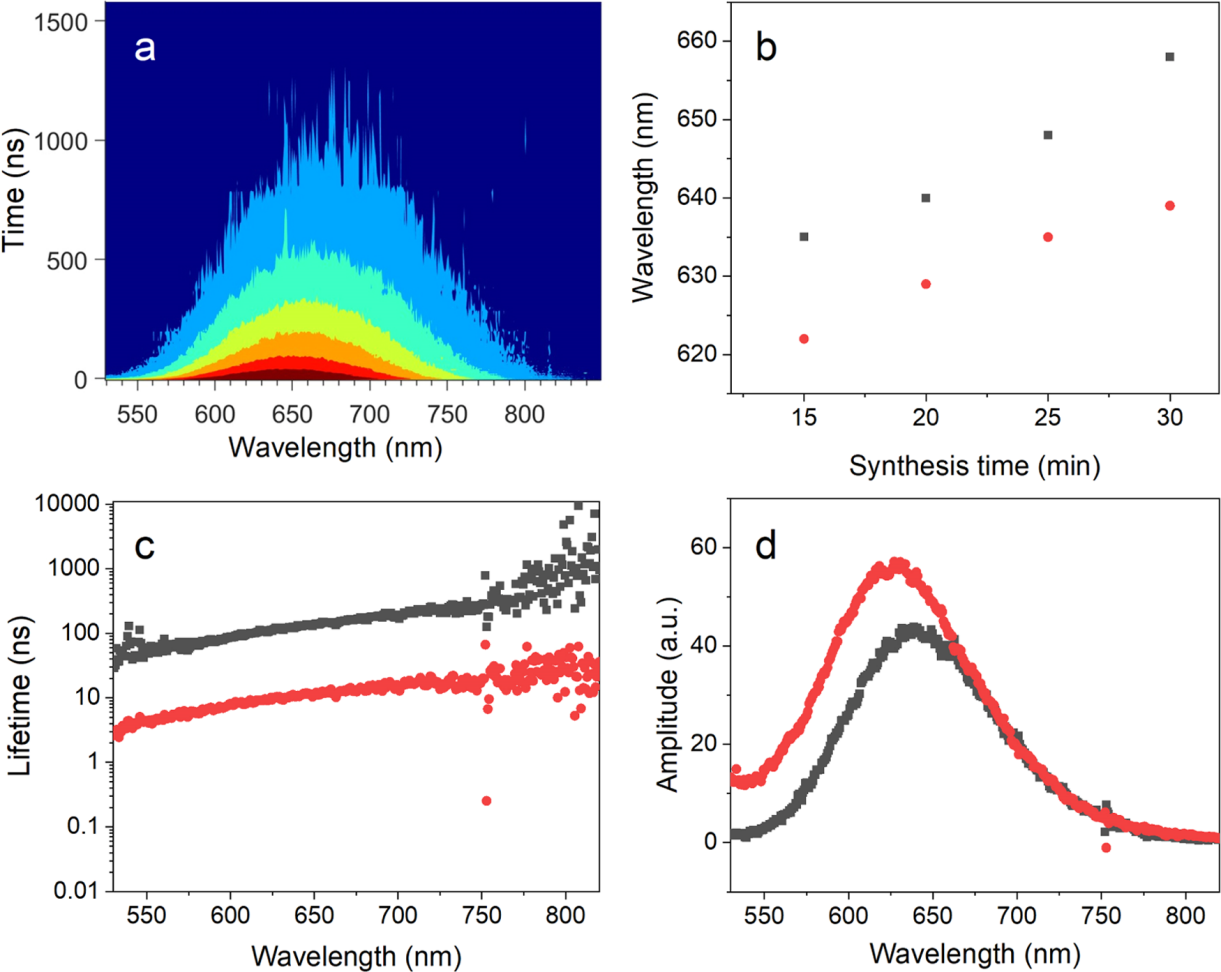
Kinetics of photoluminescence decay: (a) Photoluminescence decay spectrum over the first 1500 ns after excitation at 450 nm (sample with a 20 min synthesis time, 1.9 nm particles). A satisfactory fit was achieved with two components. (b) Size dependence of the peak position for the amplitude of each component (shorter component in red, longer component in black). (c) Fitted lifetimes of the two components and (d) spectra of their amplitudes for the 1.9 nm particles. These components correspond to band gap emission (red circles) and volume defect emission (black squares).

Figure 6a shows a typical PL decay spectrum for nanoparticles with a synthesis time of 20 min (size = 1.9 nm). Spectra for samples with synthesis times of 15, 20, 25, and 30 min were all similar to a broad peak and increasing red shift for larger nanoparticles, consistent with steady-state PL measurements, while larger particles were prone to aggregation during time-resolved experiments and thus were not included in further analyses. The PL emission peak shifts to longer wavelengths also with increasing delay time before decaying to zero within ∼1500 ns (Figure S1 in the Supporting Information). This suggests the existence of a shorter lifetime component(s) and a longer-lived, red-shifted component, as observed for stoichiometric CIS NPs previously.^28^

Indeed, the decay of fluorescence had to be fitted by two exponentials with lifetimes on the order of ∼10 and ∼100 ns to obtain a satisfactory fit (Figure S2 in the Supporting Information) for all sizes of nanoparticles. Both lifetimes were wavelength-dependent, increasing with the fluorescence wavelength (Figure 6c). Therefore, the data could not be analyzed by a global analysis^63^ and instead were fitted locally at each wavelength. Figure 6d shows the fitted amplitude of both components as a function of wavelength. For all sizes of nanoparticles, a short wavelength fast component and a longer wavelength slowly decaying component were observed. PL maxima for both components shifted to longer wavelengths with increasing synthesis time (and particle size, Figure 6b), reflecting a decrease in quantum confinement with increasing particle size.

Figure 6c shows the wavelength dependence of the fitted lifetimes. For all particle sizes, the values varied between approximately 5 and 20 ns for the fast component and ∼50 and 250 ns for the slow component. An average lifetime was obtained by averaging over the 550–750 nm range. The average lifetimes of the two components were found to be independent of particle size, with overall average values of 11.6 ± 0.6 and 150 ± 6 ns for the short and long components, respectively. Values for the fitted lifetimes are shown in Table 1, together with the corresponding peak positions (λ_*max*_) for each component. The area fractions (Table 1) of the two fitted components did not depend on the size. This suggests that the relative contribution and the two recombination pathways do not change with increasing particle size. Due to the larger separation between an electron and a hole, the defect (surface) states have usually longer lifetimes than the intrinsic band exciton recombination. In addition, these defect states also have lower energy. Therefore, the longer, red-shifted PL component was attributed to defect emission, and the shorter, blue-shifted PL to recombination within the first excited band.

**Table 1:** Synthesis Time, Nanoparticle Size, Average PL Lifetimes, Maxima of Their Amplitude Distribution (λ_PLmax_) and Area Fractions (*A*_xfrac_), and Cu/In Ratio for Particles with Synthesis Times between 15 and 30 min

**synth. time (min)**	**NP size (nm)**	**τ**_**1**_**(ns)**	**λ**_**1max**_**(nm)**	***A***_**1frac**_	**τ**_**2**_**(ns)**	**λ**_**2max**_**(nm)**	***A***_**2frac**_	**Cu/In**
15	1.8 ± 0.2	11.2 ± 0.4	622 ± 1.5	0.61 ± 0.04	137 ± 4	635 ± 2.1	0.39 ± 0.04	0.18 ± 0.01
20	1.9 ± 0.3	13.0 ± 0.4	629 ± 0.2	0.57 ± 0.01	167 ± 5	640 ± 0.2	0.47 ± 0.01	0.22 ± 0.01
25	2.0 ± 0.3	11.9 ± 0.3	635 ± 0.6	0.56 ± 0.01	149 ± 4	648 ± 0.5	0.44 ± 0.01	0.19 ± 0.01
30	2.5 ± 0.4	10.2 ± 0.3	639 ± 0.4	0.58 ± 0.01	148 ± 4	658 ± 0.3	0.42 ± 0.01	0.32 ± 0.02

### Transient Absorption Spectroscopy

**Figure 7:**
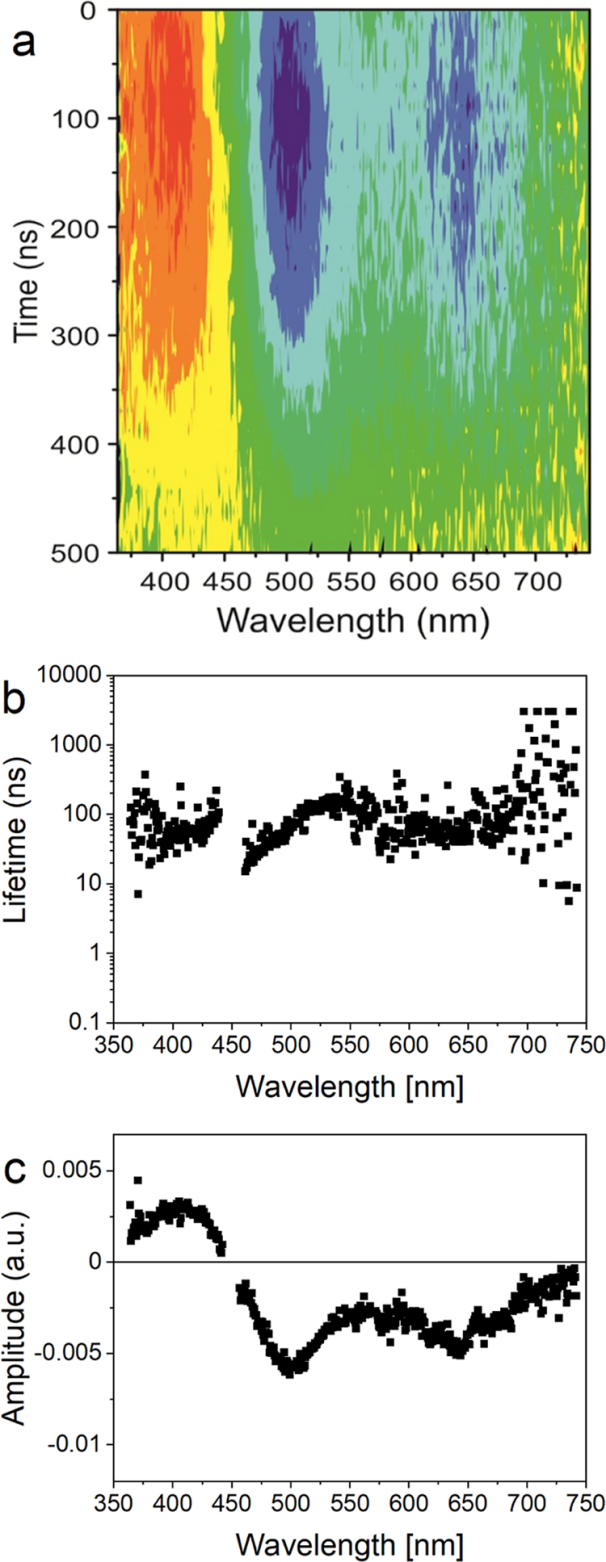
Transient absorption after excitation at 450 nm (sample with a 20 min synthesis time, 1.9 nm particles). The scattered laser radiation was omitted. (a) Transient absorption spectrum over the first 500 ns after excitation. (b) Lifetime dependence on wavelength. (c) Amplitude dependence on wavelength. The first excited band and the band attributed to surface states are well discernible as negative bands with minima at ∼500 and ∼650 nm, respectively. The lifetimes are increasing within the first excited band with wavelength, while they are approximately constant for the surface states.

The low PLQY means that the main recombination pathway is nonradiative. To investigate the kinetics of these processes, we employed transient absorption spectroscopy (pump–probe) with ns resolution. Figure 7a shows transient absorption spectra for QDs with a synthesis time of 20 min (size 1.9 nm) collected after excitation at 450 nm (see also Figure S3 in the Supporting Information for TA spectra at discrete delay times). The transient absorption exhibits three regions that decay on a 10–150 ns time scale (Figure S3 in the Supporting Information). The positive transient absorption at wavelengths shorter than the excitation wavelength represents excited-state absorption. The negative signal in the 450–550 nm region maps within the first excited-state band and thus predominantly represents ground-state bleaching due to the population of excited states. The weaker negative feature at longer wavelengths above 600 nm falls within the luminescence band, both from the band gap and the defect states. This transient absorption signal is assigned to ground-state bleaching due to the defect states, in addition to stimulated and spontaneous emission from all of the states. The data were fitted locally as in the case of PL decay, but a single lifetime was sufficient to describe the decay at each wavelength (Figure 7b). Figure 7c shows the amplitudes of the kinetics. The first excited band and the band attributed to surface defects (and the band gap luminescence) are discernible as negative bands with minima at ∼500 and ∼650 nm, respectively. This interpretation is further supported by the wavelength dependence of the lifetimes in the 450–550 nm region. The values increase with the wavelength from ∼30 to ∼150 ns, as they reflect the lifetimes of nanocrystals with increasing size. The lifetimes are approximately constant (∼65 ns) above 600 nm, which is typical for defect states.

**Figure 8:**
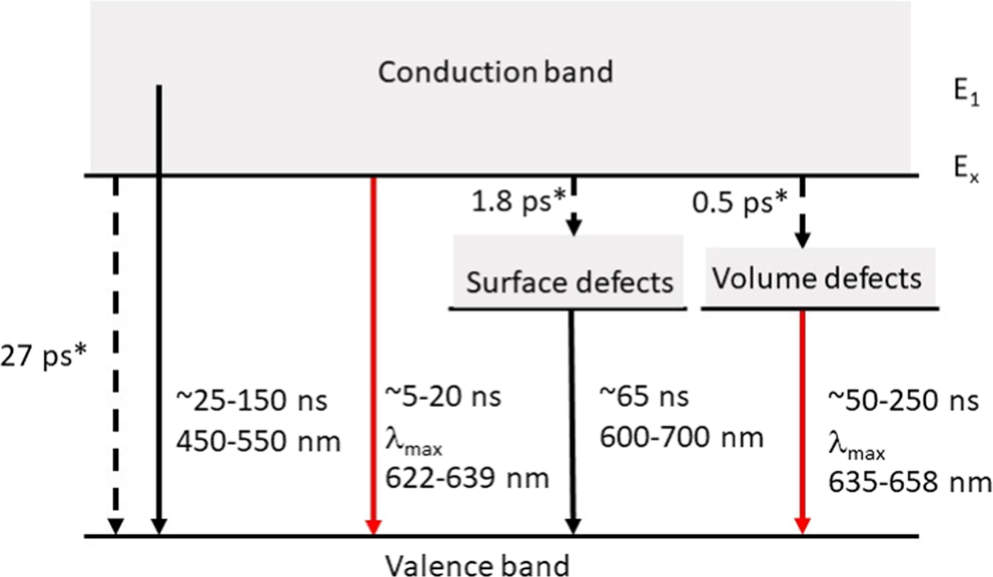
Schema of energy levels explaining the observed recombination pathways. Most of the relaxation occurs nonradiatively (black arrows) and only 2–6% radiatively (red arrows). The nonradiative relaxation was wavelength-dependent within the first excited state, while approximately constant values were observed for the surface states. The blue-shifted band gap emission decays 1 order of magnitude faster than the red-shifted defect emission. Both sets of radiative relaxation are wavelength-dependent. The previously delineated fast picosecond nonradiative processes are shown as dashed lines^52^ (relaxation times marked with *).

Previously, it was shown by transient absorption that core–shell CIS-ZnS nanoparticles exhibit picosecond excitation relaxation from the conduction band (Figure 8) to the ground state and into various intragap states that mediate further radiative and nonradiative recombination on a slower time scale.^52^ In comparison with the core–shell particles, the core-only CIS nanoparticles exhibit much lower PLQY, and hence, the fast picosecond relaxation from the conduction band (not measured here) is expected to dominate nonradiative recombination. Here, we found a slower (25–150 ns lifetime) nonradiative recombination from the first excited state and radiative recombination (wavelength-dependent lifetime of 5–20 ns) from the band gap edge states (Figure 8). Interestingly, both the PLQY and copper contents are approximately proportional to the synthesis time and nanocrystal size. This suggests that the dominant nonradiative recombination route is linked to copper deficiency and that these defects are located on the particle surface, while the photoluminescence is linked to recombination at copper sites.^64^ Indeed, we detected states with energy below the conduction band (PL and TA spectrum red-shifted from the band gap edge, i.e., 600–700 nm) that relax via nonradiative recombination (constant lifetime of 65 ns determined from TA) or radiatively (PL wavelength-dependent lifetime of 50–250 ns). These might be the two distinct sub-band-gap states that were detected previously (Figure 8).^52^

## Conclusions

In conclusion, we have demonstrated a novel method for the direct synthesis of high-quality hydrophilic nanoparticles with PLQY comparable to the best-reported values for core CIS nanoparticles ideally suited for direct coupling to biological molecules in light harvesting and charge transfer applications. Their small sizes further contribute to their colloidal stability in aqueous solutions. We have also delineated the dominant radiative and nonradiative excitation decay pathways (Figure 8). Most of the relaxation occurs nonradiatively, with wavelength-dependent lifetimes within the first excited band with approximately constant lifetimes for the defect states. This is consistent with published work,^28^ which also observed two key recombination pathways contributing to PL emission, one within the first exciton band and the second for the defect states. Through careful control of nanoparticle size and synthesis of deliberately copper-deficient nanoparticles with the degree of copper deficiency controlled by synthesis time, we have been able to shed new light on the role of size and composition on the optical properties of these nanoparticles. While both size and Cu/In ratio have profound effects on the nanocrystal properties, it remains to be seen whether these two parameters can be separately controlled.

The defect-associated energy levels, which exhibit nanosecond lifetimes, are tunable within the 650–750 nm spectral region and thus overlap with the energy levels in photosynthetic light-harvesting complexes (LHCs). LHCs contain closely packed pigments (chlorophyll), often with delocalized excited states (strong excitonic coupling), and support fast energy transfer.^65^ A close proximity of LHCs (i.e., < 1 nm) to a core-only QD may lead to further excitonic coupling to the surface-associated energy levels,^66^ resulting in fast (fs–ps time scale) energy transfer.^67,68^ This mechanism is distinct from the Förster-type transfer, which relies on weaker transition dipole interaction and is well established for core–shell QDs and synthetic or biological dyes.^69^ In fact, the wider-band-gap material that is usually used for coating core–shell nanocrystals would prevent such a strong excitonic coupling. Thus, the preparation of tunable core-only CIS nanocrystals as described here constitutes a prerequisite for developing hybrid materials that exploit strong excitonic coupling between QDs and self-assembled photosynthetic pigments.^70^

## Experimental Section

### Materials

Indium acetate (99.99%), copper iodide (99.999%), mercaptoundecanol, thiourea, and ethylene glycol were purchased from Sigma-Aldrich. HS-PEG(3400)-Biotin was purchased from ChemQuest. Methanol (analytical reagent grade) was purchased from Fisher. All chemicals were used as supplied.

### Synthesis of QDs

QDs were prepared via precursor thermal decomposition. Briefly, 24 mg (0.125 mmol) of copper iodide, 73 mg (0.25 mmol) of indium acetate, 38.2 mg (0.5 mmol) of thiourea, 200.4 mg of mercaptoundecanol, and 64.7 mg of Biotin-PEG(4300)-SH (molar ratio of 1:50 thiol/biotin-–thiol, corresponding to on average to approximately 3–4 biotin-thiols per QD) were mixed in 4 mL of ethylene glycol. After purging with argon, the flask was heated under reflux to 170 °C while stirring. Aliquots for analyses were taken using a glass syringe at 10, 15, 20, 25, 30, 40, 50, and 60 min (timed from the start of heating) and quenched by injecting into room-temperature methanol, effectively stopping the reaction while keeping the products dispersed. Due to larger sample requirements, in the case of QDs prepared for X-ray diffraction, aliquots were not taken, and instead, the reaction was quenched at 15 min by submerging the reaction vessel in cold water.

### Nanoparticle Cleaning

Unbound and unreacted compounds as well as the residual ethylene glycol were removed using a 30 kDa KrosFlow mPES hollow fiber filter module in conjunction with a KrosFlo Research IIi Tangential Flow Filtration System set at a flow rate of 60 mL min^–1^. The pressure of the sample entering and leaving the membrane tube was 5.9 and 4.1 psi, respectively. The pressure applied on the outside of the membrane was 0.1 psi providing a transmembrane pressure of 5.2 psi. Due to the negative transmembrane pressure, the dispersing solvent was able to pass through the membrane, thereby removing the excess reagents, byproducts, unbound ligands, and residual amounts of ethylene glycol. The volume of the sample within the system was allowed to reduce by half before being made up to its initial volume with fresh methanol. This was repeated 5 times, leading to a >96% reduction in sample contamination. QDs were stored in the dark at 4 °C until use.

### TEM and EDX

Nanocrystal size and morphology were examined using high-resolution transmission electron microscopy (HRTEM) with an FEI Tecnai TF20 field emission gun (FEG)-TEM operated at 200 kV and a Gatan Orius SC600A CCD camera. QDs dispersed in methanol were deposited onto gold-coated carbon grids by drop casting. Regions of interest were determined using low-magnification images (<120,000×). Size distributions of QDs were determined using either 220,000 or 390,000 magnification images, based on a sample of on average 127 from each aliquot. Lattice spacings were determined from high-magnification (690,000×) images using a 2D Fourier transform. Elemental composition was established by energy-dispersive X-ray analysis using an Oxford Instruments 80 mm X-Max SDD EDX detector on areas identified from low- and medium-magnification images as having a high density of nanoparticles.

### XRD

Owing to larger sample requirements, an additional synthesis was performed as described above without aliquots. The synthesis was quenched at 15 min by immersing the reaction vessel in cold water. The entire reaction product (∼100 mg) was used. Solvent exchange into pure (18.2 MΩ) H_2_O was performed using the KrosFlo Research IIi Tangential Flow Filtration System under the conditions described above using H_2_O instead of methanol to make up the lost volume. QDs in H_2_O were frozen using liquid nitrogen and lyophilized to produce a fine powder. The XRD pattern was obtained using a Panalytical Model X’Pert Pro MPD X-ray diffractometer with a Cu Kα source (λ = 0.154 nm) and an X’cellerator detector. A continuous scan over a 2θ range from 10 to 80° was performed with an acquisition time of 45 min.

### XPS

The elemental stoichiometry of QDs was determined using a Thermo Fisher ESCA Lab 250 with a monochromated aluminum Kα X-ray source. The incident X-ray beam had an energy of 1486.69 eV with a rectangular illumination profile of ∼300 by 700 μm. Measurements were made under high vacuum (*p* < 1 × 10^–9^ mbar). QDs in methanol were drop-cast onto ozone-cleaned fragments of microscope slides with a 50 nm evaporated gold layer on 5 nm of chromium. Samples were immobilized onto stainless steel sample holders by means of double-sided carbon tape. Low-resolution survey spectra were obtained using a pass energy of 160 eV over a binding energy range of −5 to 1200 eV with 0.5 eV increment. High-resolution spectral scans were obtained by using a pass energy of 20 eV with 0.05 eV increments. An acquisition time of 240 s was used for both the survey and high-resolution scans. Binding energies were calibrated by setting the 1s carbon peak to 285.0 eV. Copper-to-indium ratios were determined using the areas under the Cu 2p_3/2_ and In 3d_5/2_ peaks.

### Spectroscopy and Spectrofluorophotometry

UV–visible absorption spectra were recorded by using a Genesys 6 spectrophotometer. Photoluminescence spectra were obtained using a Shimadzu RF-5301PC Spectrofluorophotometer. 450 nm excitation and emission slit widths of 5 nm were used. Samples used for UV–visible absorption spectroscopy were diluted so that the optical density at the first transition was ∼0.1. Band gap energies were determined from Tauc plots.^71^ Samples used for steady-state fluorescence measurements were diluted to fall below the linear response region of the spectrofluorometer.

### PLQY Measurements

Absolute values for PLQY were determined by using a spectroscope equipped with a 10 cm diameter integrating sphere (SphereOptics GmbH). Sample excitation was via indirect diffused light from LEDs emitting in the range of 2.4–3.1 eV. Light from the integrating sphere was coupled to a silica fiber bundle, the output of which was imaged by an Acton SpectraPro SP2150i spectrometer with a Spec-10:400B back-illuminated CCD camera. PLQY was determined as the ratio of the number of emitted and absorbed photons. A more detailed theoretical discussion and further details on the setup can be found elsewhere.^72^

### PL Lifetime Analysis

Time-resolved nanosecond PL lifetime measurements were carried out using a setup consisting of an imaging spectrometer (iHR-320, Horiba) and an intensified CCD camera (PI-MAX 512RB, Princeton Instruments) with a 2 ns gate width. An optical parametric oscillator (PG122, EKSPLA), pumped by a Q-switched Nd:YAG laser (NL303G, EKSPLA) operating at 10 Hz, was used as the excitation pulse source. The pulse length was about 3 ns, and its energy was set to ∼0.25 mJ at 450 nm. Spectra were measured over the ∼500 to ∼900 nm range up to ∼1.6 μs after excitation and averaged from 50 to 200 measurements at any given delay. A delay generator (DG535, Stanford Research Systems) was used to control the time delay between the excitation pulse and the detection gate. The sample was kept at 4C during the experiments to minimize its degradation. Steady-state absorption and PL spectra were measured before and after the experiments to check whether no degradation occurred during acquisition. The PL decays were fitted with a sum of exponentials convoluted with an instrument response function, which was assumed to be a Gaussian. The data were fitted independently at each wavelength, using the same set of initial parameters.^73^ Two exponentials were sufficient to fit the PL decays at all wavelengths (see the Supporting Information for more details).

### Transient Absorption Spectroscopy

Nanosecond transient absorption spectroscopy was performed using the same setup as PL measurement with the addition of a xenon flash lamp (FX-1161, PerkinElmer) to generate a spectrally broad light pulse used as probe and reference beams. Measurement was run at a 10 Hz frequency with the excitation laser operating at 5 Hz. Transient absorption between ∼350 and ∼750 nm was calculated from the intensities of the probe and reference beams measured with and without excitation. Spectra were measured at 34 delays nonlinearly sampling the first 500 ns after excitation. The sample was kept at 4C to minimize degradation. Steady-state absorption and PL spectra were measured before and after the experiments to check whether no degradation occurred during data acquisition. The fitting was done in the same way as for the PL data (see the Supporting Information for more details), but one lifetime was sufficient to fit the data.

## Supporting Information Available

Time-resolved data analysis; transient photoluminescence spectra at selected delays; photoluminescence kinetics at selected wavelengths; and transient absorption spectra at selected delays (PDF)
