# Supplementary material for: Direct Synthesis and Characterization of Hydrophilic Cu-Deficient Copper Indium Sulfide Quantum Dots
Source: ACS Omega. 2024 Apr 4;9(15):17114–24. doi: 10.1021/acsomega.3c09531 (PMC11025077; doi:10.1021/acsomega.3c09531)
Supplement: Supplementary file 1 — ao3c09531_si_001.pdf [file ao3c09531_si_001.pdf]

Supplementary information

Direct synthesis and characterisation of hydrophilic Cu-deficient copper indium sulphide quantum dots

Amanda Richardson[1,2,6], Jan Alster[3], Petro Khoroshyy[3,7], Jakub Psencik[3], Jan Valenta[3], Roman Tuma[1,2,5]*, Kevin Critchley[1,4*]

[1]Astbury Centre for Structural Molecular Biology, University of Leeds, LS2 9JT, U.K.

[2]School of Molecular and Cellular Biology, Faculty of Biological Sciences, University of Leeds, LS2 9JT, U.K.

[3]Department of Chemical Physics, Faculty of Mathematics and Physics, Charles University, Prague, Czech Republic

[4]School of Physics and Astronomy, Faculty of Engineering and Physical Sciences, University of Leeds, LS2 9JT U.K.

[5]Faculty of science, University of South Bohemia, Ceske Budejovice, Czech Republic.

*corresponding authors

[6]Present address: University of Sheffield, Sheffield, U.K.

[7]Present address: Institute of Organic Chemistry and Biochemistry of the Czech Academy of Sciences, Czech Republic

Time-resolved data analysis

The data were fitted by a sum (over *i*) of exponentials convoluted with the instrument response function assumed to by Gaussian:

$$f(t,\lambda) = \sum_i \{a_i(\lambda)\exp(-t/\tau_i)\times H\} \otimes \{N \times \exp[-(t-t_0)^2/2\sigma^2]\}$$

where *a* is the wavelength (λ) dependent amplitude of the exponential, *t* is time, τ is the lifetime, *H* is the Heaviside step function (which determines the begging of the decay, time zero), *N* is the normalization factor of the Gaussian and σ and t_0 are Gaussian parameters of the laser pulse (centre and variance). The data were fitted independently at each wavelength. Two exponentials were sufficient in the case of photoluminescence, one component in the case of transient absorption.

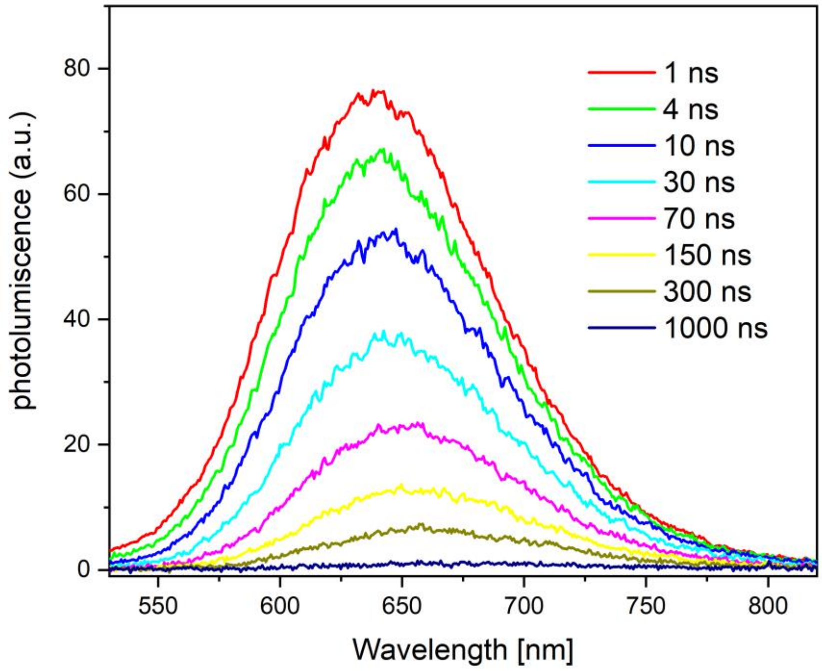

Fig. S1 Transient photoluminescence spectra at selected delays. The spectra show an increasing red shift of the transient absorption maximum with the delay.

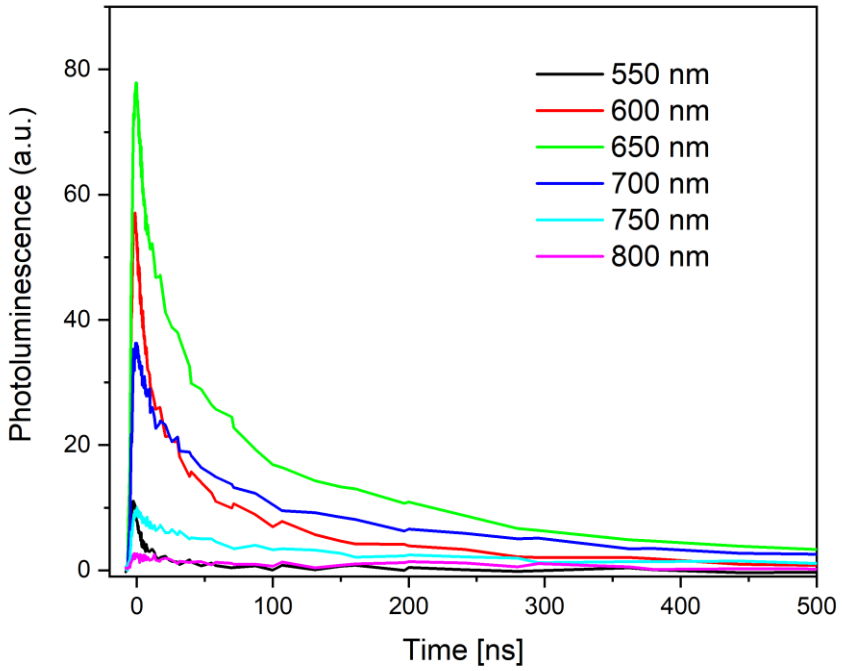

Fig. S2 Photoluminescence kinetics at selected wavelengths. The kinetics show an increasing relative contribution of the slower decay component with the wavelength.

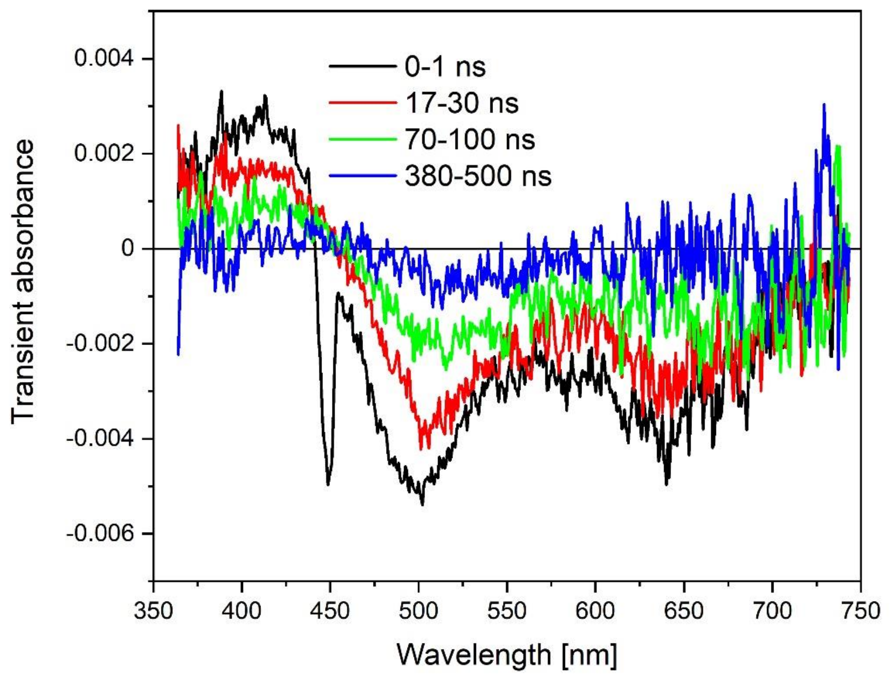

Fig. S3 Transient absorption spectra at selected delays. The spike at 450 nm at the 0-1 ns delay is caused by the scattered signal from the excitation laser pulse. The transient signal was rather weak under the applied experimental conditions ($\Delta A<0.006$). To improve the signal-to-noise ratio, each transient spectrum is presented as an average from the data within the indicated range.
